# Sense-Making, Mutual Learning and Cognitive Shifts When Applying Systems Thinking in Public Health – Examples From Sweden Comment on "What Can Policy-Makers Get Out of Systems Thinking? Policy Partners’ Experiences of a Systems-Focused Research Collaboration in Preventive Health"

**DOI:** 10.34172/ijhpm.2020.106

**Published:** 2020-07-05

**Authors:** Monica E. Nyström, Sara Tolf, Helena Strehlenert

**Affiliations:** ^1^Department of Learning, Informatics, Management and Ethics (LIME), Medical Management Centre, SOLIID, Karolinska Institutet, Stockholm, Sweden.; ^2^Department of Epidemiology and Global Health, Umeå University, Umeå, Sweden.; ^3^Stockholm Gerontology Research Centre, Stockholm, Sweden.

**Keywords:** Systems Thinking, Soft Systems Methodology, Healthcare Development, Double-Loop Learning, PolicyMakers, Sweden

## Abstract

It is widely acknowledged that systems thinking (ST) should be implemented in the area of public health, but how this should be done is less clear. In this commentary we focus on sense-making and double-loop learning processes when using ST and soft systems methodology in research collaborations with policy-makers. In their study of policymakers’ experiences of ST, Haynes et al emphasize the importance of knowledge processes and mutual learning between researchers and policy-makers, processes which can change how policy-makers think and thus have impact on real-world policy concerns. We provide some additional examples from Sweden on how ST has been applied to create learning and shared mental models among stakeholders and researchers in national and regional healthcare development initiatives. We conclude that investigating and describing such processes on micro-level can aid the knowledge on how to implement ST in public health.

## Introduction

In the past decade there has been an increased interest in how systems thinking (ST) can be applied to public health. As opposed to reductionist approaches with limited sets of targeted interventions, ST considers the complexity of a phenomenon and its context, eg, interventions being interdependent of each other and the environment, an approach that could benefit addressing wicked problems.^1-3^ In ST complex interventions are viewed as systems in themselves, interacting with other parts of the system and setting off reactions that can be unpredictable and unintended.^4^ Best et al^5^ have identified understandings and abilities needed to use ST in practice: an understanding of how the system is organized, managed and lead; an understanding of and an ability to manage system stakeholders and networks; an ability to conceptualize, model and understand dynamic change; and being able to manage content and infrastructure of explicit and tacit knowledge while understanding the role of information flows in change processes. Despite the growing interest, the understanding of ST and its use within public health remains unclear.^6^ Moreover, there are relatively few applied studies focusing on ST in public health and the need for further studies and practical applications has been highlighted.^7,8^

Within ST, a distinction is made between hard systems and soft systems methodologies (SSM). In a recent review, Carey et al^7^ conclude that the qualitative and action-oriented SSM techniques can be more useful within the public health domain. In SSM, concepts such as sub-systems, transformations, and stakeholders are used as metaphors for communicating about complex situations and phenomena. SSM is designed to tackle real-world problems, which may be difficult to define, to uncover the worldviews of system actors and to facilitate learning.^9^ Developing shared cognition and team mental models among key actors involved in change processes is important to achieve systems change.^10^ Team mental models can aid the formulation of collective expectations and explanations of tasks that the team is facing, enhance shared problem representation, and facilitate communication and coordination of team activities.^11^

Haynes et al^12^ provide an empirical study of practical use of ST by investigating an Australian research collaboration that uses a ST approach, ie, the Australian Prevention Partnership Centre (hereafter called the centre). More specifically, their qualitative, explorative paper focuses on policy-makers’ experiences of engaging with ST in various cross-sector research collaborations advocated by the centre.

In this commentary we aim to further discuss the challenges in studying the use of ST in practice, and the embedded sense-making processes that are central in real-world change settings where divergent perspectives are common. We provide empirical examples of how we have used SSM techniques to enhance the development of shared mental models in previous research partnerships.

## Australian Policy-Makers’ Experiences of System-Focused Collaborations

Haynes et al investigate the policy-makers’ reasons for participating in the center’s system-focused collaborations, their views and experiences of ST and, indirectly, the potential effect of ST on their work. The center used three general approaches to ST in their collaborations with policy-makers. Firstly, by organizing research projects that are informed by systems concepts but based on other disciplines for methodology (ie, implementation, scaling-up, and economic and program evaluation). Secondly, by using systems methods, ie, systems dynamics, social network analysis, causal loop diagrams and SSM in applied research projects. Thirdly, by focusing on systems capacity building in tailored development projects. However, detailed information on how ST was implemented in the various projects is lacking, which makes it difficult to fully understand the processes that influenced the policy-makers’ experiences. For example, the reported variation in experiences may be due to individual or organizational factors, or to variation in how ST was applied.

Most of the policy-makers saw benefits in ST and the collaboration opportunities offered. The system-focused collaboration seemed to generate a conceptual shift in worldviews among some policy-makers that in turn affected multiple aspects of their work. The ST approach used influenced the overarching strategic direction or thinking across a program, while system tools exerted a lesser influence. Haynes et al conclude that the knowledge processes may be more important than the knowledge products, highlighting mutual learning between researchers and policy-makers. The authors report a process of thinking-together-in-practice, which is in line with the reflective and problem-solving process in action learning.^13-15^ Such processes can generate ‘double-loop learning’^16^ with a shift in worldviews, and also a transformation of implicit knowledge into explicit knowledge, which seem to be important aspects of implementing ST. Double-loop learning recognizes that how a problem is defined and solved can in fact be a source of the problem, and that changing the basic assumptions (rather than to keep using existing goals, decision-making rules and action strategies) may be necessary. Focusing on learning, development of team mental models among policy-makers may be imperative to a better understanding on how to implement and study ST in real-world settings.

## Experiences of Using SSM in Research Collaborations in Sweden

Here we provide examples of ST approaches that we used for achieving double-loop learning and shared mental models in research projects focusing on the implementation of healthcare policies and regional development programs. The examples are based on our experiences of working with national and regional policy-makers and strategic actors on several system levels in health policy initiatives addressing wicked problems over the last decade (Table).

**Table:** Health Policy Initiatives and References to More Information

**Research Partnerships**	References
*Regional initiative 1. 2007 – on-going program* A regional health promotion program for children 0-18 years (primary prevention) involving primary healthcare, dental care, social care, pre-schools and schools	^18-20^
*Regional initiative 2. 2009–2014* Development and implementation of a regional strategy for improving the capability for continuous organizational improvement and learning within elderly care and care of people with functional impairments (provided by the nine municipalities in the region)	^21-23^
*National initiative 3. 2012–2014* A national initiative, Better Life for the ill elderly people, in order to improve care provided by the regions, municipalities and private and not-for profit care providers	^24-26^
*National initiative 4. 2015-2022* A national initiative to improve delivery care and women’s health in all of Sweden’s 21 regions (primary healthcare and hospital care)	On-going project (publications in Swedish)

We have participated in these national and regional initiatives as research collaborators. In this role we have been able to follow the initiatives in real-time, collect data continuously and to present and discuss observations with policy-makers and key actors, in order to support the process and to strengthen the impact of the initiatives.^17^ SSM was used both to facilitate sense-making as part of the development and the research process. In line with using ST as a metaphor and tool for uncovering worldviews we have worked together with partners to visualize situations, processes and structures, to construct maps and models for enhancing shared knowledge and team mental models.

**Figure 1:**
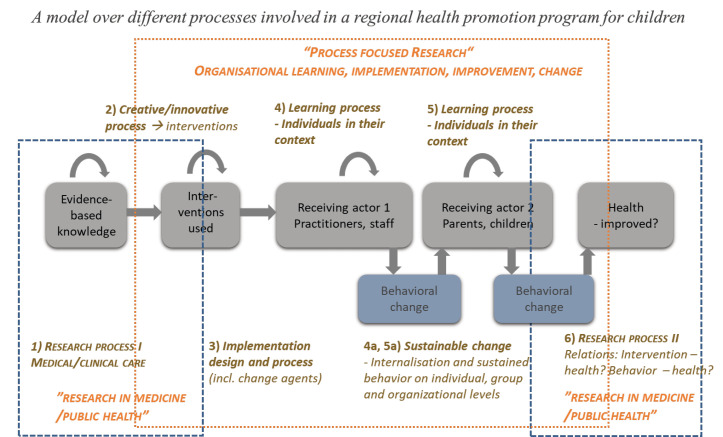
A Model of the Change Process in a *Regional Health Promotion Program for Children* and the Involved Actors’ Perspectives.

Our first example stems from a *regional health promotion program for children* where we collaborated with strategic actors in the region’s child healthcare and public healthcare units, including public health researchers, development functions, and managers. A model to enhance the shared understanding of the program and the actors’ various perspectives (ie, worldviews) is presented in Figure 1. This model was created to envision the change aimed for and the process to achieve it, as well as the multiple research interests connected to the initiative. It was developed during a series of meetings where initially disparate goals and perspectives, indicating differences in worldviews, were expressed. Based on the initial discussions we drew a crude model that was adapted and fine-tuned successively by the group. The modelling process unveiled both potential challenges and benefits of using ST and combining different perspectives, and it helped us prevent and resolve situations that otherwise could have resulted in power struggles or conflicts.

**Figure 2:**
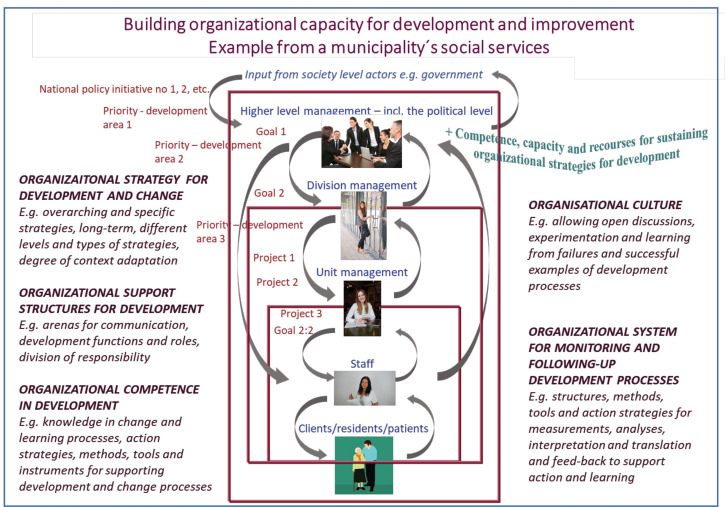
A Model of the Main Features Involved in a Regional Strategy to Build Organizational Capacity for Development, Improvement and Learning.

Our second example (Figure 2) was developed in an action-research project focused on the development and implementation of a* regional strategy for improving the capability for continuous organizational improvement and learning* (Table). The project involved a partnership with a regional R&D unit responsible for the development of elderly care and care of people with functional impairments in the region’s municipalities. We started by interactively mapping current change initiatives at the different levels of the system and to explore the concept of organizational capacity for development and learning. This was visualized in a feedback-loop structure depicting the organizational context of decision-makers and managers in the municipalities (Figure 2). The model enabled us to jointly figure out and discuss action strategies for various stakeholders, including ourselves as research collaborators. Since it first crude form we have also used this model in relation to two national initiatives (Table) and adapted its features to fit other parts of the welfare system and the changes aimed for in these initiatives.

These are two of many examples of visual representations that we have created together with research partners to enhance learning and shared mental models in multiple collaborative research projects over the past decades.^17,18,21^ Our approach shares features with Foster-Fishman and colleagues’^27^ framework for transformative systems change. In our collaborative projects we attempt to understand and visualize the different perspectives concerning the current problem situation and identify system parts and patterns to locate root causes to problems. The overarching aim is to aid stakeholders to explore and accommodate differences among competing worldviews, in order to enrich their understandings of the actual problems or change situations.

## Discussion

ST approaches can be useful for identifying and understanding patterns in systems, and the interest for applying ST to the field of public health is increasing. However, the understanding and the use of these ideas in the public health literature is still poor.^6^ Traditionally, research in public health has been more interested in causes and effects of single interventions than the processes involved in creating change. Contextual and cognitive aspects of learning and behavioural change, and approaches used to influence them, has not been given as much focus. Haynes et al identify knowledge and learning processes as fundamental and highlight the possibilities of using ST and collaborative approaches when working with policy-makers. They conclude that “*changing how we think*offers the greater potential for policy impact because it transforms people’s mental models and the principles by which they carry out their work” (p. 8). Consequently, how this cognitive shift can be achieved deserves special attention.

This highlights one of the main challenges of implementing ST in public health – that of changing people’s cognition and behavior. Such change requires a deep, ‘double-loop’ learning process^16^ that can be demanding for both individuals and groups. It is important for researchers and policy-makers to understand this cognitive change process, so that they in turn can support practitioners in making sense of policy intentions and changing their views and practices accordingly.^28^ For ST to be useful for policy-makers a closer analysis of their views on how policy-induced change is understood and how ST and knowledge of the system (eg, organization theory) and targets groups (eg, behavioral psychology) can benefit the development of healthcare and public health.

However, there are specific methodological demands when studying changes and processes longitudinally in complex systems.^29^ The complexity and the many levels and perspectives to adhere to for researchers and policy-makers in the Australian and Swedish examples can make it challenging to gather both process and outcome data and describe the strategies used to make ST transform from theoretical descriptions to practical use.

To aid development we also need to know more about the barriers for using ST for key actors in different parts of the health system, including researchers. The challenges might differ, and ST support needs to be adapted to the particular country, system, culture and situation. Moreover, not only individuals may have to change worldviews, organizational systems might need to become more agile in order to act on external inputs in new ways.^30^

Any attempt to make ST permeate the development and improvement of any country’s healthcare system can be over-whelming and the process needs to start somewhere. The partnership centre is an interesting national intervention, which has the potential to aid the implementation of ST. This empirical case will hopefully be further studied with regards to details of the use of ST and the collaboration with policy-makers. It is especially interesting to know more about the learning process and the effects on actors’ worldviews, their approaches to problem-solving and development. Using SSM to enhance sense-making and mutual learning in our partnerships has so far aided collaboration and the knowledge-development process – and affected the way involved actors think, in line with the findings in Haynes and colleagues’ study.

Developing ST and putting it to practice in public health requires collaboration and is essentially a learning process for policy-makers and practitioners as well as for researchers. This process involves testing of various perspectives, questioning of basic assumptions about how the system works and re-framing of mental models. Focus on public health interventions and their effects can easily overshadow the details of how to support their implementation. Another important consideration is to discuss how the health system is viewed, its structure, boundaries and relations to how health can be improved. Such consideration opens up for a wider perspective for policy-makers on how health systems relates to the Sustainable Development Goals on global, regional, national, and local levels.^30^

Our experiences of using ST resonate with Haynes et al regarding the importance of building a solid foundation for collaborative work and to promote ST among policy-makers, even though it most likely will be both challenging and time-consuming.

## Ethical issues

Not applicable.

## Competing interests

Authors declare that they have no competing interests.

## Authors’ contributions

All authors contributed to the writing of the manuscript and all approved the final manuscript.

## Authors’ affiliations

^1^Department of Learning, Informatics, Management and Ethics (LIME), Medical Management Centre, SOLIID, Karolinska Institutet, Stockholm, Sweden. ^2^Department of Epidemiology and Global Health, Umeå University, Umeå, Sweden. ^3^Stockholm Gerontology Research Centre, Stockholm, Sweden.
